# Looking back to move forward: 50 years of epidemiology of lymphohaematopoietic malignancies for prevention, and healthcare planning in Sardinia, Italy

**DOI:** 10.1177/03008916251384524

**Published:** 2025-10-18

**Authors:** Giorgio Broccia, Pierluigi Cocco, Jonathan Carter, Cansu Ozsin-Ozler, Sara De Matteis

**Affiliations:** 1 Department of Haematology and Bone Marrow Transplants, Hospital A. Businco, Cagliari, Italy; 2 Centre for Occupational and Environmental Health, Division of Population Health, Health Services Research & Primary Care, University of Manchester, Manchester, UK; 3 Research Centre for Fluid and Complex Systems, University of Coventry, Coventry, UK; 4 Hacettepe University, Faculty of Dentistry, Department of Paediatric Dentistry, Ankara, Turkey; 5 Department of Health Sciences, University of Turin, Italy

**Keywords:** Epidemiology and prevention, haematology-oncology, forecasts, haematology care planning

## Abstract

In previous reports, we showed the diversity in the epidemiological features of the most prevalent lymphohaematopoietic malignancies (LHM) in Sardinia, Italy. In this paper, we reviewed those findings, aiming to: 1) explore the geographic correlation between the various LHM; 2) estimate the standardised incidence rates up to 2017; 3) compare our estimates with nationwide trends; 4) project such trends up to 2030; and 5) predict the expected LHM cases in the upcoming years by health district to assess the adequacy of the existing Haematology and Healthcare services. We observed a high probability for all LHM combined exceeding the expectation in the central-northern and central-eastern areas of the region. There was a weak, though significant, geographic correlation between non-Hodgkin lymphoma (NHL) and multiple myeloma (MM), but not the other LHM. The estimated incidence of all LHM was higher in Sardinia than nationwide. The chances for the existing Haematology units and Healthcare services to adequately match the expected incident cases appear low. This paper concludes a 50-year journey in the epidemiology of LHM among a genetically peculiar population. We hope our results will promote appreciation of the value of extending the routine registration of incident cancer cases for prevention and healthcare planning.

## Introduction

The Italian region of Sardinia is the second largest and one of the least populated islands in the Mediterranean Sea. While renowned for its stunning coastline, scientists know it better for the unique genetic features among the local population,^1^ which have been preserved through millennia of isolation and selective pressure exerted by malaria.

Such unique genetic features have provided fertile ground for genetic studies on longevity,^2^ multiple sclerosis,^3^ and type I childhood diabetes.^4^ Being aware of these genetic peculiarities and the healthcare opportunities offered by the routine registration of incident cancer cases, more than 50 years ago, one of the coauthors (GB) initiated an exhaustive database of incident cases of lymphohaematopoietic malignancies (LHM) among the regional population. We validated this database by comparing the relative risk in small geographic areas with those obtained from mortality and hospitalisation data.^5^ We also compared the absolute number of cases and rates with Cancer Registry data for the overlapping years (1993-2003) in the northern and central regional provinces.^6^ Based on these comparisons, we could confidently define our database as population-based and use it to describe LHM time trends and their geographical spread over the region. Our results showed linear upward trends for childhood leukaemia, non-Hodgkin lymphoma (NHL), multiple myeloma (MM), chronic lymphocytic leukaemia (CLL), acute myeloid leukaemia (AML), and myelodysplastic syndrome (MS). Hodgkin’s lymphoma (HL) and acute lymphoblastic leukaemia (ALL) exhibited peculiar age-related differences, the first with a significant upward trend below 45 years, but not among the population aged 45 years or more, and the second showing a phase contrast oscillatory pattern at age below 25 *vs.* above 65 years. We also confirmed previously reported clusters of childhood leukaemia, a peculiar geographic distribution of NHL in the northern-central areas of the region, and previously undetected clusters of HL among the female population, and AML among the male population.^6-12^

Interest in the Sardinian LHM database was motivated by the limited coverage of the local population by Cancer Registries, which was also delayed by two-decades. Additionally, repeated alarms in the local media about excess LHM cases in specific areas were speculatively associated with hypothetical environmental risk factors.^5^

In this paper, we reviewed the LHM space-time coordinates and their association with potential environmental risk factors, compared the time trends between LHM, evaluated their geographic correlation, and compared regional with nationwide time trends. We also explored the expected incidence in the upcoming years to verify whether the judgment about the appropriate size and location of the existing Haematology units and Health Services, expressed in a preliminary analysis using data collected up to 1993,^13^ would continue to be valid.

## Methods

Details on the database of incident LHM in Sardinia have been previously provided.^13^ Briefly, the database comprises records of 14,744 cases of LHM diagnosed region-wide from 1974 to 2003; records are anonymous and grouped by gender, 10-year age-group, residence, diagnosis, and year of diagnosis. The senior haematologist who initiated the database (GB) reviewed the available clinical and pathological information for each case, which provided consistency over the years and the various diagnostic centres. The database completeness was previously validated.^5,6^

### Statistical methods

#### Incidence and time trend

We calculated the age- and sex-standardized incidence rate of non-Hodgkin lymphoma (NHL, International Classification of Diseases-10th Revision (ICD-10) codes: C82-C88, C91.4), MM (ICD10 code C90), HL (ICD10 code: C81), and leukaemias (all lymphatic and myeloid, acute and chronic, forms combined, ICD10 codes: C91-95.9 excluding C91.4 and C94.4) along the study period for the total population, and men and women separately, using the World Health Organization world population as the standard, for each of the 356 administrative units existing in Sardinia as of 1974. We incorporated 21 centres that became independent in subsequent years into the administrative unit of origin for consistency over time.

For this paper, we lump all leukaemia types to allow the comparison with international databases, such as the International Agency for Research on Cancer’s Globocan database.^14^

We assumed that the ratio between the 1992-2003 incidence rates reported in the two Sardinian Cancer Registries and the region-wide estimates from the database during the overlapping period remained constant over time. Then, we applied that ratio to the Cancer Registry rates to estimate the regional LHM incidence for each quinquennium from 1992 to 2017 and compared these estimates with those for Italy from the International Agency for Research on Cancer (IARC) Globocan database.^14^ We chose the Globocan database instead of available alternatives^15^ as it is based on real data from the existing Cancer registries, and therefore, its estimates can be considered an unbiased sample of the total population.

#### Spatial distribution

The Bayesian method we applied to calculate the geographical distribution of the posterior probability of NHL, MM, HL, and leukaemias over the 356 Sardinian communes was also previously described.^1^ Briefly, to avoid the problem of a large number of small administrative units and the consequent large chance variation of the ratio between observed and expected incident cases, we calculated the likelihood ratio between the probability, given the expected prior (P1), of exceeding the pre-determined 0.999 threshold in the Poisson distribution of the observed cases *vs*. the probability of not exceeding it (P0). The likelihood ratio was plotted in a map of the administrative units’ territorial borders using a chromatic scale of increasing darkness (white *p* ⩽ 0.165, light grey *p* = 0.166-0.335, medium-light grey *p* = 0.336-0.50, medium-dark grey *p* = 0.501-0.80, dark grey *p* = 0.801-0.95, black *p* ⩾ 95%). The maps are publicly available on the Italian Institute for Statistics (ISTAT) website (https://www.istat.it/it/archivio/104317) under the Creative Commons BY 3.0 IT License. We also explored the reciprocal spatial correlation between the four LHM groups with Spearman’s correlation analysis. In evaluating the results from this analysis, we applied the Bonferroni correction to the 5% *p*-value threshold to reject the null hypothesis.^16^

For each administrative unit, we extracted from multiple sources data on economic deprivation, distance from the nearest hospital, type of residence, geology, background radiation, proximity to industrial and military settlements and cork farms, and size of livestock breeding relative to the resident population, and calculated the relative risks for NHL, MM, HL and leukaemias and their respective 95% confidence interval associated with increasing levels, with the lowest category, the null category, or the quaternary marine deposits in the case of geology as the reference, using multivariable Poisson regression analysis, adjusting by age and sex.

#### Future trends and assessment of the adequacy of the existing haematological care units

For each LHM group, we used the Joinpoint software, developed by the Statistical Methodology and Applications Branch of the US National Cancer Institute, freely available online,^17^ to calculate the 1992-2017 annual per cent change (APC) and its 95% confidence interval. Then, based on the 1974-2003 age-standardised rates and the 1992-2017 regional APC, we plotted the expected incident cases up to 2030 for each local health district in the region.

The Ethics Committee of the University Hospital of Cagliari approved the use of the 1974-2003 LHM database for scientific research purposes (protocol N. PG 2019/18070, 18 December 2019) in agreement with the Code of Ethics of the World Medical Association (Declaration of Helsinki).

## Results

### Time trends in the LHM incidence

**Figure 1.**
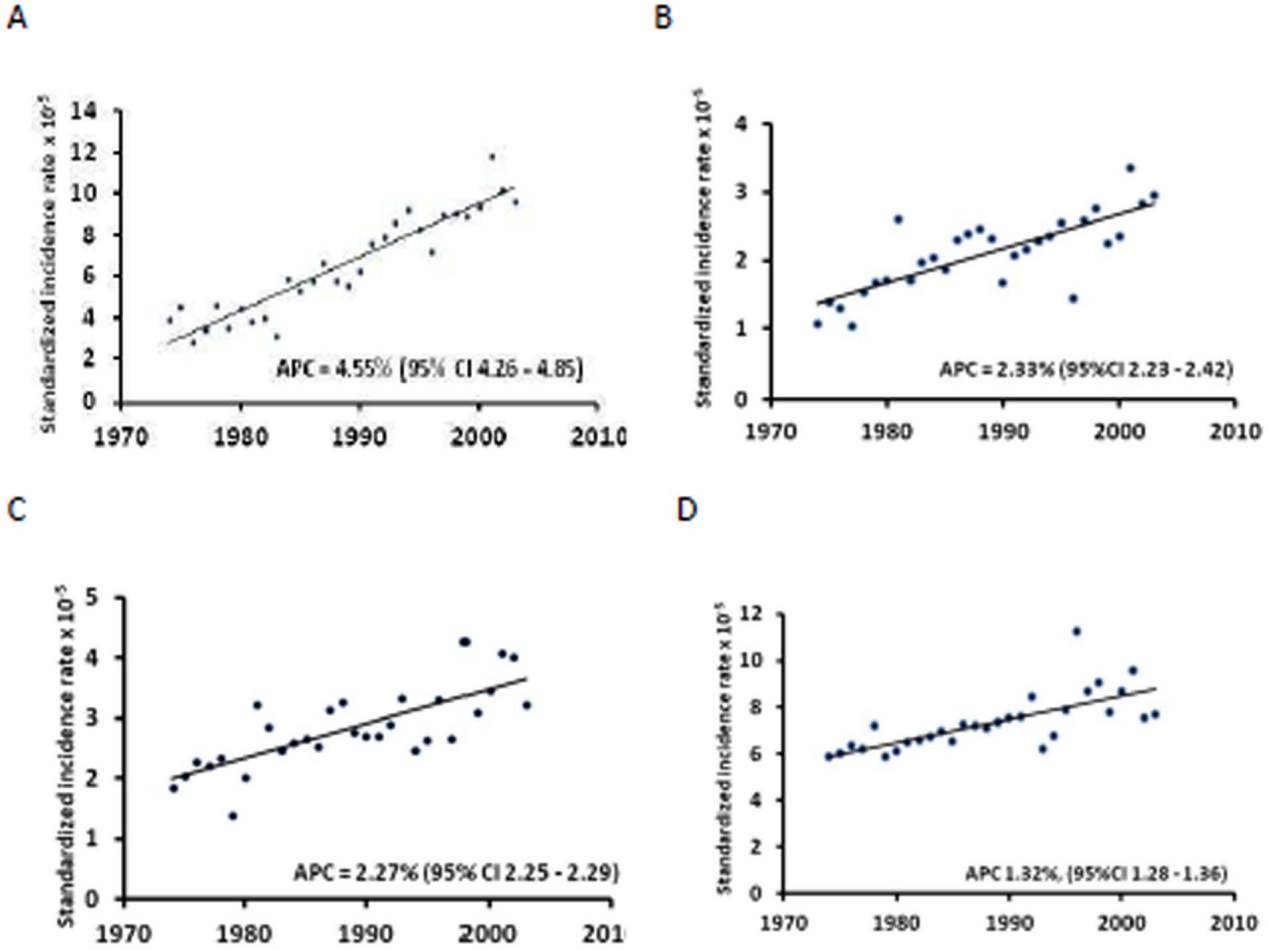
1974–2003-time trend of the standardised incidence rate x 10^-5^ of LHM groups in the total population of Sardinia, Italy. A: NHL, B: MM, C: HL, D: leukaemias.

The 1974-2003 LHM incidence in Sardinia varied by histological group, suggesting a varying response to modifiable external factors (Figure 1). NHL and MM showed sharp upward trends consistent in both sexes and all age-groups.^6,8^ The annual per cent change (APC) was 4.55% (95% CI 4.26 – 4.85) for NHL and 2.33% (95% CI 2.23 – 2.42) for MM. HL also showed a significant upward trend (APC = 2.27%, 95% CI 2.25 - 2.29) in both sexes,^12^ which differed by age at diagnosis, with a sharp upward trend below age 45 years and stable rates from age 45 onwards.^12^ Overall, the incidence of leukaemias also increased over time (APC = 1.32%, 95% CI 1.28, 1.36), mostly related to an linear increase in CLL incidence,^8^ while ALL incidence differed by age,^11^ and that of myeloid leukaemias also increased for all forms combined and for AML and MS, but not for chronic myeloid leukaemia (CML).^10^

**Figure 2.**
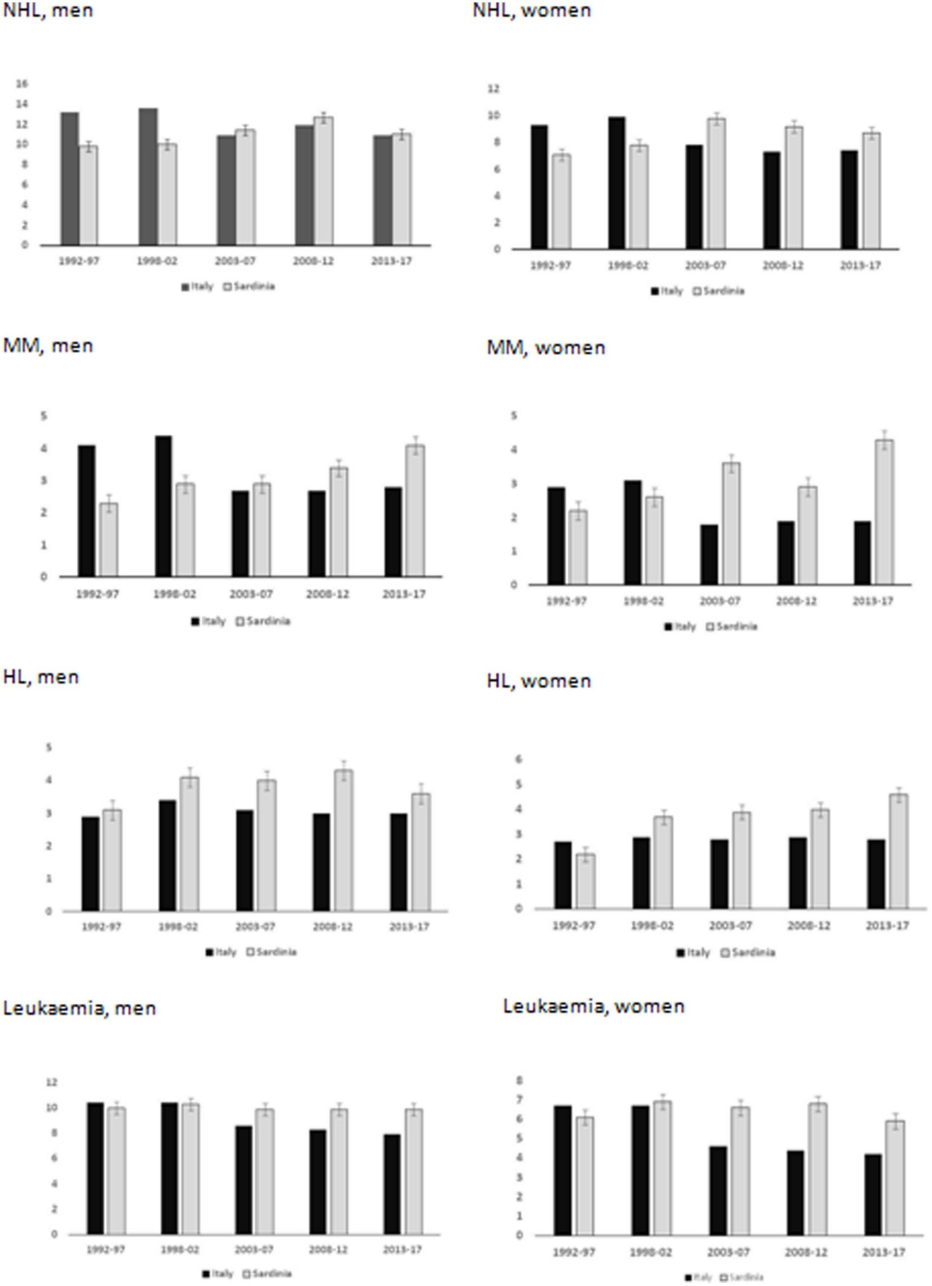
Estimates of 2004-17 LHM incidence in Sardinia compared to Italy.

As shown in Figure 2, NHL incidence decreased in Italy from 1998 onwards in both sexes, while in Sardinia it continued increasing for another decade and decreased in 2013-2017. Over the total period, the APC was 10.7% (95% CI 2.0 – 23.4). MM continued to increase in both sexes among the Sardinian population (APC = 8.5%, 95% CI 5.9 – 11.2), contrasting the decreasing incidence observed in Italy from 2003 to 2007 and the stable rates in the subsequent years. HL incidence in Sardinia (APC = 2.4%, 95% CI -4.1 – 9.0) also behaved differently from nationwide, with increasing rates that continued in women and levelled off in 2013-2017 among men. Leukaemia incidence did not vary from 1992 to 2017 (APC = -1.2%, 95% CI -3.3 – 1.0), contrasting the sharper nationwide decreasing trend.

### Space distribution of LHM

**Figure 3.**
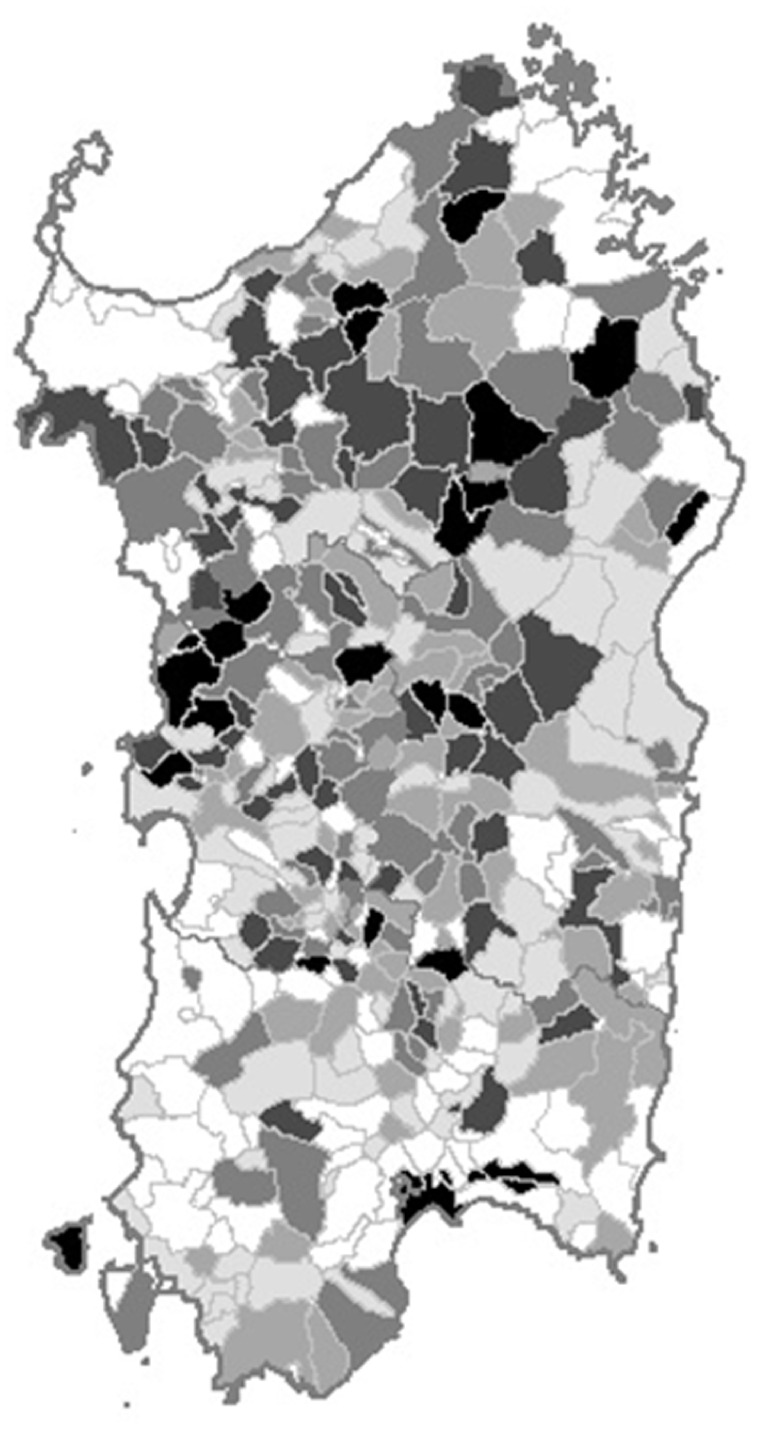
Map of the 1974-2003 probability of a high incidence of lymphohaematopoietic malignancies by administrative unit in Sardinia, Italy.

The purpose of investigating the overall LHM impact by administrative unit was twofold: 1) to identify administrative units where unknown factors might have increased LHM susceptibility. This analysis could allow identifying administrative units at risk undetected by the analysis of specific LHM, because of the small number of events; and 2) to assess whether the territorial distribution of the specialist haematology units and health services matched the oncohaematology healthcare needs. As shown in Figure 3, 17 administrative units, located mainly in the north-eastern and central-western areas of the island, had a high probability of exceeding the prior threshold of LHM cases above expectation.

Table 1 shows the correlation matrix between the 1974-2003 probabilities of excess cases of the individual LHM in the 356 Sardinian administrative units. Due to the observed differences in time trend, ALL below or above age 25 years and HL below or above age 45 years were separately evaluated. NHL showed a weak, though significant correlation with MM and HL diagnosed at age ⩾ 45 years; CLL showed a weak correlation with myeloid leukaemia and acute lymphoblastic leukaemia among children and young adults (age ⩽ 24 years), which in turn correlates with HL diagnosed at age ⩽ 44 years. Lastly, HL probabilities at ⩽ 44 and ⩾ 45 years showed a weak correlation.

**Table 1.** Correlation matrix of the probability of excess cases between the most prevalent lympho-haematopoietic malignancies.

	NHL	MM	ML	CLL	ALL⩽24	ALL⩾25	HL⩽44	HL⩾45
NHL	1.000	0.175*	0.041	0.033	−0.043	0.045	0.029	**0.111**
MM		1.000	0.089	0.077	−0.053	0.087	0.024	0.072
ML			1.000	**0.154**	0.023	0.066	−0.066	−0.021
CLL				1.000	0.029	**0.130**	0.017	0.012
ALL⩽24					1.000	0.092	**0.123**	0.022
ALL⩾25						1.000	−0.050	0.002
HL⩽44							1.000	**0.133**
HL⩾45								1.000

ML, myeloid leukaemia (acute and chronic forms combined); ALL⩽24, acute lymphoblastic leukaemia at age ⩽ 24 years; ALL⩾25, acute lymphoblastic leukaemia at age ⩾ 25 years; HL⩽44, Hodgkin’s lymphoma at age ⩽ 44 years; HL⩾45, Hodgkin’s lymphoma at age ⩾ 45 years. The correlation coefficients significant at P<0.05 are in bold. The * indicates those that remained significant after applying the Bonferroni correction.

### Environmental exposure and socio-economic factors

Table 2 graphically summarises the association between hypothetical environmental risk factors and the risk of specific LHM. In this analysis, ALL cases were jointly considered independent of the age at diagnosis. Urban residence was associated with the risk of most LHM, apart from MM and myeloid leukaemia (acute and chronic forms combined).^6-12^ CLL risk increased 9% for residents 30 km or more from the nearest hospital,^9^ while the HL risk decreased by 10% for residents 14 km or more from the nearest hospital.^12^ Living in the most deprived administrative units resulted in a lower risk of NHL,^6^ as was the case for myeloid leukaemia risk among those whose probability of indoor radon exceeding 300 Bq/m^3^ was 31% or more.^10^ Interestingly, residing in administrative units with a high prevalence of goat breeding was consistently protective against NHL, myeloid leukaemia, ALL, and HL,^6,10-12^ while residing in the surroundings of industrial areas resulted in an excess risk of ALL among adults.^11^ Finally, cork harvesting was a risk factor for CLL,^9^ while the risk of MM was slightly lower.^8^ However, very few administrative units had cork harvesting as a major component of their economy, and the number of observed events was small, generating a higher probability of chance findings.

**Table 2.** Summary results of the association between potential environmental risk factors and the risk of individual LHM (red indicates a positive association; green indicates an inverse association).

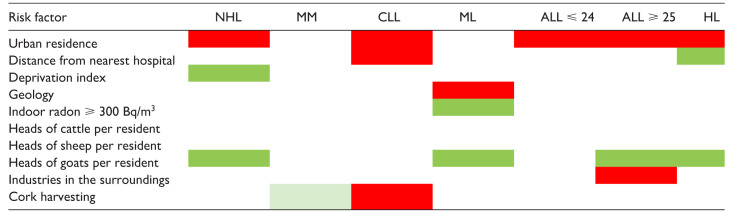

ML, myeloid leukaemia (acute and chronic forms combined); ALL⩽24, acute lymphoblastic leukaemia at age ⩽ 24 years; ALL⩾25, acute lymphoblastic leukaemia at age ⩾ 25 years.

### Future trends and adequacy of the existing health services

**Figure 4.**
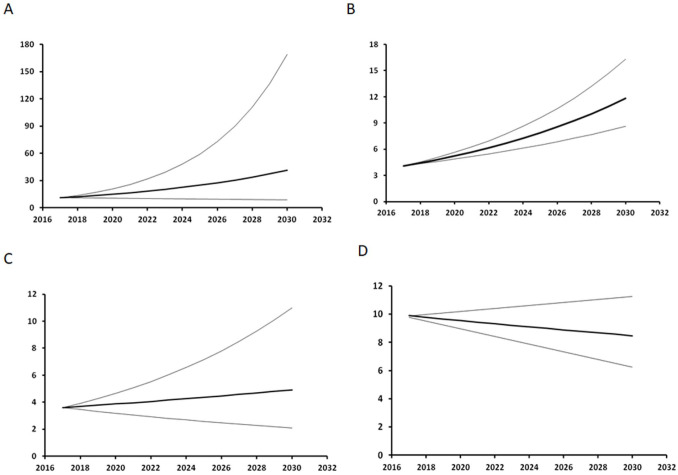
Estimated future LHM trends in the LHM incidence rates among the general population of Sardinia, Italy. A: NHL, B: MM, C: HL, D: Leukaemia. The thin lines represent the 95% confidence interval.

As shown in Figure 4, the future trends for NHL and HL to 2030 remain uncertain. However, in both cases, a moderate increase in incidence is the most likely scenario. In contrast, MM incidence shows a clear tendency to a further increase, while the opposite trend is suggested for leukaemias. Table 3 shows the estimated expected cases of NHL, MM, HL, and all leukaemias combined per year by health district up to 2030, and matches the expected cases with the currently available health services to understand whether these might efficiently respond to future care demands. Using the same criteria as in the previously mentioned 2005 paper,^13^ each existing Haematology unit for adult patients in Sardinia can treat 130-150 new cases annually, for a total of 390-450 cases region-wide, excluding rarer haematological forms of borderline or uncertain neoplastic nature, not considered in our analysis. However, according to our projections, 697 cases (95% CI 421 – 1,375) will occur annually, indicating that only the most optimistic figure, associated with a 2.5% probability, supports the statement that the existing haematology units are appropriately sized to match future healthcare demands.

**Table 3.** 2017-2030 expected annual cases of NHL, MM, HL, and leukaemia, and existing health services by health district in Sardinia, Italy.

Health District	NHL	MM	HL	Leukaemias	Hospital facilities
Alghero	9 (3.9 - 24.4)	5 (3.9 - 5.6)	4 (2.3 - 5,6)	6 (5.4 - 7.2)	++
Ozieri	5 (2.0 - 12.4)	2 (1.8 - 2.6)	1 (0.9 - 2.2)	3 (2.2 - 2.9)	++
Sassari	26 (11.1 - 69.0)	16 (13.1 - 19.3)	9 (6.0 - 14.4)	17 (14.5 - 19.1)	+++
La Maddalena	2 (0.8 - 5.0)	1 (0 - 0.6)	1 (0.5 - 0.8)	1 (0.5 - 0.7)	+
Olbia	12 (4.8 - 30.0)	9 (7.6 - 11.1)	3 (2.2 - 5.4)	7 (6.1 - 8.1)	+++
Tempio	4 (1.4 - 9.1)	2 (1.6 - 2.3)	1 (0.9 - 2.1)	3 (2.8 - 3.6)	+
Ogliastra	6 (2.4 - 15.1)	3 (2.6 - 3.7)	2 (1.4 - 3.5)	5 (4.1 - 5.4)	++
Nuoro	13 (5.5 - 34.2)	6 (5.0 - 7.4)	3 (2.3 - 5.4)	7 (6.5 - 8.6)	+++
Siniscola	4 (1.6 - 10.1)	2 (1.9 - 2.8)	1 (0.6 - 1.4)	2 (2.1 - 2.8)	-
Sorgono	2 (0.9 - 5.9)	2 (1.3 - 1.9)	1 (0.9 - 2.1)	2 (1.6 - 2.1)	+
Ales-Terralba	5 (1.9 - 12.1)	3 (2.6 - 3.8)	2 (1.1 - 2.6)	4 (3.7 - 4.9)	-
Oristano	8 (3.1 - 19.6)	5 (4.2 - 6.1)	3 (1.9 - 4.6)	7 (5.9 - 7.8)	++
Planargia	4 (1.8 - 11.2)	3 (2.5 - 3.7)	2 (1.1 - 2.6)	4 (3.6 - 4.7)	+
Marghine	3 (1.4 - 8.5)	2 (1.5 - 2.2)	1 (0.8 - 1.9)	2 (1.6 - 2.2)	+
Guspini	5 (2.2 - 13.9)	4 (3.5 - 5.1)	2 (1.4 - 3.5)	4 (3.6 - 4.9)	++
Sanluri	5 (2.0 - 12.6)	4 (3.4 - 4.9)	2 (1.1 - 2.6)	4 (3.5 - 4.6)	-
West Cagliari	12 (4.9 - 30.6)	8 (6.2 - 9.1)	4 (2.3 - 5.6)	14 (12.4 - 14.9)	+++
Metropolitan Cagliari	35 (14.5 - 90.3)	18 (15.0 - 22.0)	11 (7.2 - 17.3)	26 (22.6 - 29.9)	+++
Quartu - Parteolla	13 (5.4 - 33.5)	7 (5.7 - 8.4)	6 (3.6 - 8.6)	11 (9.6 - 12.6)	+
Sarcidano - Trexenta	5 (2.0 - 12.3)	3 (2.6 - 3.9)	2 (1.2 - 2.9)	4 (3.4 - 4.5)	+
Sarrabus - Gerrei	2 (0.9 - 5.2)	2 (1.7 - 2.6)	1 (0.4 - 0.9)	2 (1.6 - 2.2)	+
Carbonia	6 (2.4 - 15.1)	3 (2.4 - 3.4)	2 (1.2 - 2.9)	5 (4.6 - 6.1)	++
Iglesias	5 (1.9 - 11.9)	3 (2.7 - 3.9)	2 (1.2 - 2.9)	5 (4.0 - 5.3)	++
St. Anthiocus-St. Peter Islands	2 (0.9 - 5.3)	2 (1.4 - 2.1)	1 (0.6 - 1.4)	2 (1.9 - 2.5)	-
Sardinia (total)	367 (153 - 959)	117 (96.9-142)	67 (44.1 - 106)	146 (127 - 168)	

+++ major general hospitals and presence of specialist haematology units; ++ general hospital; + minor hospital; - no hospital.

## Discussion

From exploring the past LHM trends, geographic spread, and environmental correlates in Sardinia, Italy, we learned that LHM are a more complex universe than the diagnostic categories that classify them.

### Time trends

Time trend differences suggest a diverse susceptibility to the same external risk factors or susceptibility to diverse risk factors. Between 1974 and 2003, among the Sardinian population, we observed upward trends for most LHM but not leukaemias. We estimated that the linear increase in MM incidence will continue up to 2017, contrasting the nationwide flat pattern during the same years and in the future. It is unclear what would be driving this upward trend. As Broccia et al. suggested in 2005, at least up to those years, the observed upward trend might have been accounted for by substantial diagnostic improvements and better access to specialised haematology care, particularly among the elderly population.^13^ However, for the trend to continue in subsequent years, at odds with nationwide observations, some unknown external factors may have contributed.

Among Sardinian men, NHL levelled off about ten years later than nationwide and about five years later among women, with broad confidence intervals around the 1992-2017 central APC estimates. Therefore, trend projections up to 2030 reflect this uncertainty with wide, diverging confidence intervals of the regression coefficients. We previously observed that administrative units with a high probability of excess NHL cases between 1974 and 2003 also experienced a high incidence of COVID-19 in the first year of the pandemic, when vaccines were not yet available.^18^ We speculated that the most diffuse NHL subtypes and COVID-19 might share some common mechanisms; however, based on those findings, we cannot establish a link between past SARS-Cov-2 infection and the risk of some specific NHL subtypes.

In our analyses of myeloid malignancies, myelodysplastic syndrome showed the most impressive upward trend,^10^ which started after the publication of the French-American-British Working Group diagnostic criteria in 1976.^19^ It was most likely explained by the resulting increase in the diagnoses of a disease previously unrecognised as malignant. Future studies should focus on the high-risk area we identified in north-western Sardinia, where the trend’s slope for myeloid malignancies was twice as steep as in the low-risk areas.^10^

For the 1974-2003 period, we could explore the time trend for the major leukaemia subtypes.^9-11^ A peculiar oscillatory pattern characterised the temporal evolution of ALL below age 25 years, and flat rates for cases diagnosed among adults and the elderly.^11^ Such a difference in the shape of the time trend would suggest that two different forms of the disease may affect youngsters and the elderly, with the first but not the second associated with an external agent that spreads among the population, affects the most susceptible, and then vanishes because of increase in immunity and/or adapting mutations (in case of infectious agents) or environmental improvements (in case of non-infectious agents). For the subsequent years, we relied on Cancer Registry data to create our estimates and the Globocan database to compare them to the nationwide rates. Both sources combine all leukaemias, which resulted in losing diagnostic precision to preserve the possibility of a comparison. The most frequent form, i.e. chronic lymphocytic leukaemia, showed increasing trends between 1974 and 2003.^8^ Acute, but not chronic myeloid leukaemia, also showed an upward trend (APC 3.18%, 95% CI 2.99 – 3.37). However, our estimates for the near future suggest a downward trend for all leukaemias combined. As previously suggested,^13^ increasing access to laboratory exams for various purposes may have serendipitously detected CLL cases in otherwise asymptomatic subjects. Spurious upward trends might have resulted from progressive improvements in healthcare access unconfirmed in the subsequent years. Interpretation of these estimates requires caution, as relying on no change in the persisting trends observed up to 2017.

We observed a linear upward trend in HL incidence between 1974 and 2003,^12^ which was confirmed for diagnoses at age ⩽ 44 years, but not at more advanced ages.^12^ The hygiene hypothesis, i.e. the delay in encountering infectious agents due to hygienic improvements and the consequently weakened immune response, has been suggested as an explanation.^20^ Our estimates up to 2017 differed by sex: among women, we estimated a continuous increase, which contrasted the flat nationwide rates; among men, HL incidence increased up to 2012 and, although still higher than nationwide, decreased between 2013 and 2017. As a result, our forecast for the next five years is uncertain, as suggested by the diverging confidence interval of the regression line, with the best estimate indicating a stable or slightly increasing rate.

### Space distribution and environmental risk factors

The geographic distribution of all LHM combined among the Sardinian administrative units displays two major dark spots in the central-northern and the central-eastern areas. We previously described an excess of NHL and MM in the first^6,8^ and a clustering of high HL probability among the female population of several bordering administrative units in the second.^12^ As this area comprises small rural villages, it is possible that cases of individual LHM were too few for any excess to emerge in the analysis; their combination might have matched the statistical power requirements and allowed the excess to show up on the map. Yet, the territorial distribution of all LHM combined might have partially reproduced that of NHL, the predominant form.

We conducted a correlation analysis of the 1974-2003 probability of each of the two LHM over the 356 administrative units, which, in our intention, might suggest possible shared aetiological factors among the LHM groups, whether genetic or environmental. A few positive, though weak, correlations emerged between NHL and MM, NHL and HL at age ⩾ 45 years, CLL and myeloid leukaemia, CLL and ALL at age ⩽ 25 years, and between HL below and above 45 years. However, after applying the Bonferroni correction, only the correlation between NHL and MM held its significance. Therefore, we cannot exclude chance as the determinant of the other correlations described in Table 1.

Besides the different time trends, another element supporting LHM heterogeneity is the varying relationship with the environmental risk factors we identified (Table 2). Urban residence was associated with risk of NHL, MM, ALL diagnosed at age ⩽ 24 and ⩾ 25 years, and HL, but not MM and myeloid leukaemia. Urban residence is a surrogate for carcinogenic emissions from urban traffic, including benzene, and infectious agents transmissible through contact with the public, two major risk factors previously associated with an increasing risk of NHL, MM, and HL.^20-23^ Conversely, administrative units with a high prevalence of goat breeding showed lower risks of NHL, myeloid leukaemia, ALL at age ⩾ 25 years, and HL. Previous reports suggested an inverse association with occupational contact with goats, which, interestingly, was stronger for contacts that started early in life.^20^ Other associations emerged, including the excess CLL risk and the inverse association of HL with a more than 30 km distance from the nearest hospital, the first suggestive of difficulties in accessing proper health care for the rural elderly population, and the second being the other side of the coin of the elevated risk among urban residents. Risk of adult ALL was associated with residence in the proximity of industrial settlements; living in economically deprived administrative units was inversely associated with NHL risk; cork harvesting was a risk factor for CLL and conveyed borderline protection against MM; an elevated probability of exposure to indoor α-radiation levels from radon emissions above 300 Bq/m3 was inversely associated, and living in an area geologically consisting of metamorphic rocks was a risk factor for myeloid leukaemia. Most such findings might be due to chance. Yet, it is noteworthy that, since ancient times, the territory of Sardinia has been well known for its vast mineral deposits;^24^ depending on their concentration and type, certain minerals in some metamorphic rocks may contain trace amounts of radioactive elements such as uranium, thorium, or potassium-40, which can elevate the background gamma radiation.^25^

### Estimated healthcare burden from incident LHM in 2017-2030

Our projections up to 2030 suggest that the existing haematology units are unlikely to be sufficient to match the haematological healthcare demand in the near future. The alternative hypothesis of inadequacy has a 97.5% probability of occurring. Two strategies were previously suggested to face this likely challenge: 1) to upgrade the capacity of the existing haematology units, and 2) to create a network between those units and other existing healthcare services, including oncology, geriatrics, and medicine departments. As for the first point, besides increasing the number of beds, units of medical and nursing personnel, and equipment in the Haematology units, the existing national guidelines on the per cent bed occupancy should be applied with some flexibility in Sardinia, because of the persisting inadequacy of the transportation and communication infrastructures, the low population density (the second lowest amongst the Italian regions), and the uncertainty surrounding the estimates of future cases. These problems are currently dealt with by emergency transportation to specialised centres in mainland Italy. However, a permanent emergency is not an option for functioning healthcare systems. Regarding the second point, based on the geographic distribution of incident LHM cases, a stricter connection between local health services and the main haematology units sharing diagnostic and therapeutic protocols would facilitate patients’ access to healthcare during the clinical course of their disease.

### Limitations

An interpretative issue of environmental epidemiology results is the ecological study design and the difficulty in translating population exposure estimates to the individual level. In our previous publications, we cautioned against the so-called “ecological fallacy” in interpreting our results, particularly as it concerns the observed association with environmental risk factors.^8-12^ On the other hand, we also referred to Sir Richard Doll’s statement about the hypothesis-generating value of the time and space coordinates of neoplastic diseases, and their capability of reflecting underlying changes in the diagnostic classification and measuring the effectiveness of therapeutic advances.^26^

Another potential issue in exploring the geographical spread of LHM is the wide fluctuations of risk estimates in less populated administrative units, where the occurrence of one or two cases can generate high-risk estimates, alternating with years of absence of incident cases when the risk becomes null. This issue is especially relevant in the region of Sardinia, where 55% of the administrative units have 2000 inhabitants or less and 7% share 56% of the resident population. We are confident that our Bayesian approach effectively addressed the mixed sizes of the administrative units.

The conservative approach we used in rejecting the null hypothesis prevented several false positive results. However, our strategy might have increased the number of false negative findings. To account for this possibility, we applied a colour scale of increasing shade of grey to the administrative units’ areas according to the posterior probability of the observed cases exceeding the expectation. Then, we visually interpreted the alternation of dark and light areas in the maps.

As clarified in the Methods, our prediction of future trends was based on the 1974-2003 age- and sex-standardised rates and the 1992-2017 regional APC. Therefore, we could not consider the effect of subsequent events, such as the COVID-19 pandemic, which geographic correlation with the past NHL incidence could suggest a possible shared mechanism. Notably, the same association was not observed for HL.^12^ Therefore, the unforeseeable long-term effects of the pandemic shed a blanket of further uncertainty over our predictions.

As previously stated, our predictions of the LHM incidence up to 2030, from which we derived the number of expected cases in each health district, relied on the annual percent change from 1992 to 2017. It is unknown whether the past trends will continue or change, but we are reasonably confident that the 95% confidence interval we calculated will comprise the most likely changes.

## Conclusion

Important Public Health goals, such as identifying environmental risk factors, discovering changes in disease incidence, and properly planning the territorial distribution of Healthcare services, can only be achieved through the tedious, long-term registration and precise clinical definition of every new incident case, which is the daily routine for a Cancer Registry. Although limited to the lymphohaematopoietic malignancies, the database we used surrogated for two decades the lack of a Cancer Registry, and for another 10 years, its partial coverage. The results of our efforts suggest that a well-functioning Cancer Registry can provide better answers than the sudden outbreaks of fear towards the unknown hazards posed by any new human initiative.

This paper concludes a 50-year journey in the epidemiology of lymphohaematopoietic malignancies among a genetically peculiar population. We complemented the absence of epidemiological competence and the inadequacy of the local public health institutions with personal sacrifice and dedication. Looking back, we are confident that our results could be useful to appreciate the value of routine registration of incident cancer cases in terms of prevention and healthcare planning.
